# Genomic Profiling Reveals Synchronous Bilateral Lung Adenocarcinomas With Distinct Driver Alterations of *EML4-ALK* or *TPM3-ROS1* Fusion: A Case Report

**DOI:** 10.3389/fonc.2019.01319

**Published:** 2019-11-27

**Authors:** Benxu Tan, Xuan Jiang, Ruping Wang, Cuiping Tang, Sisi Liu, Xue Wu, Lei Xia, Xian Yu, Zhenzhou Yang

**Affiliations:** ^1^Department of Oncology, The Second Affiliated Hospital of Chongqing Medical University, Chongqing, China; ^2^Department of Research and Development, Nanjing Geneseeq Technology Inc., Nanjing, China; ^3^Translational Medicine Research Institute, Geneseeq Technology Inc., Toronto, ON, Canada

**Keywords:** bilateral lung adenocarcinomas, *EML4-ALK*, *TPM3-ROS1*, synchronous multiple primary lung cancers, next-generation sequencing

## Abstract

**Background:** *ALK* and *ROS1* rearrangement accounts for 3–6% and 1–3% of non-small cell lung cancers, respectively, while coexistence of them in the same patient is extremely rare. Only three cases have ever been reported with concurrent *ALK/ROS1* fusions in the same tumor indicating tumor heterogeneity. Therefore, comprehensive genetic profiling via next-generation sequencing (NGS) is needed to provide fully molecular diagnosis.

**Case Presentation:** A 50-year old Chinese female with resectable stage IB bilateral lung adenocarcinomas (ADCs) harbored *EML4* exon 6-*ALK* exon 19 and *TPM3* exon 8-*ROS1* exon 35 fusions in the right lower and the left upper tumors, respectively, identified by clinical NGS test targeting 425 cancer-relevant genes. The results were further confirmed at RNA level using RNA-seq. Genomic evolution analysis reveals that these bilateral tumors are synchronous multiple primary lung cancers with no shared somatic alterations for both genes and arm-level copy number variations (CNVs). No recurrence was observed during 12 months of post-surgery follow-up.

**Conclusions:** Our case is the first report of concurrent ALK/ROS1 fusions as distinct driver events of synchronous multiple primary lung cancers, and highlights the importance of individual genetic testing for each of the multiple primary tumors for fully molecular diagnosis and precise treatment decision-making.

## Background

Lung cancer is the leading cause of cancer death worldwide, and lung adenocarcinoma (ADC) accounts for nearly 50% of all lung cancers (1). Rearrangements of anaplastic lymphoma kinase (*ALK*) occur in 3–6% of ADC patients, while the incidence of c-ros oncogene 1 (*ROS1*) rearrangement is identified to be approximately 1–3% worldwide (2). Crizotinib, a multi-targeted tyrosine kinase inhibitor with high clinical efficiency, has been approved for ALK-positive or ROS1-rearranged lung cancer treatment (3). Although each of ALK or ROS1 rearrangement has been widely reported, the coexistence of ALK and ROS1 fusions in the same patient was only observed in 3 cases within a single tumor (4–6). Here we present a case for the first time with synchronous bilateral lung cancers harboring *EML4* (echinoderm microtubule-associated protein-like 4-anaplastic lymphoma kinase)*-ALK* and *TPM3* (tropomyosin 3)*-ROS1* fusion, respectively, analyzed by next-generation sequencing (NGS). Our finding gives a meaningful insight into the understanding of concurrent driver gene alterations, and highlights the importance of multiple tumor biopsies for genetic testing in patients with synchronous multiple primary tumors.

## Case Presentation

**Figure 1:**
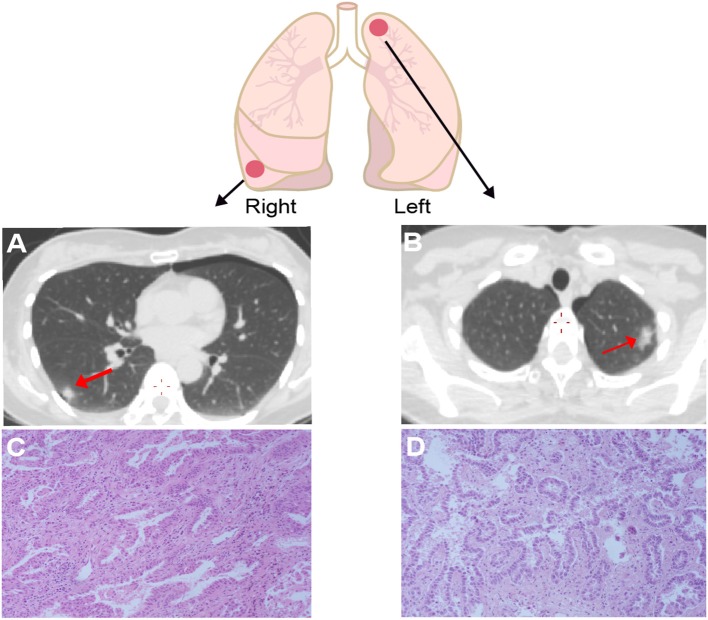
Computed tomography (CT) and Hematoxylin & eosin (HE) staining for the lesion in right lower lobe **(A,B)** and left upper lobe **(C,D)** showed both of the tumors are well-differentiated adenocarcinomas. The right one has papillary-predominant pattern, and the left one appeared with acinar-predominant and lepidic pattern. Red arrows point the tumor sites.

A 50-year old Chinese female never-smoker was diagnosed with stage IB bilateral well-differentiated lung ADCs in the right lower and the left upper lobes in August, 2018 (Figure 1). After surgical resection of the two primary ADCs, the patient was initially treated with three cycles of pemetrexed (800 mg/m^2^ d1), followed by pemetrexed 800 mg/m^2^ d1 plus bevacizumab 300 mg/m^2^ d1 for one cycle.

Given her never-smoking history, both the bilateral primary tumor tissues were subjected to target capture-based clinical NGS test for 425 cancer-relevant genes (GeneseeqPrime), and genomic alterations between the two lesions were compared (Table 1). In the right lower ADC, *EML4* exon 6-*ALK* exon 19 (*EML4-ALK*) fusion was detected at a mutant allele frequency (MAF) of 7.5%, accompanied by *DLL* point mutation (c.838G>A) and *TP53* truncation (c.1024C>T). While in the left upper ADC, *TPM3* exon 8-*ROS1* exon 35 fusion (*TPM3-ROS1*) fusion (10.8%), *DOT1L* splice site mutation (c.1924-2A>C) and *SPRED1* point mutation (c.1249A>G) were observed without any shared mutations with the other lesion (Table 1). Our results indicated that each of these synchronous primary ADCs was driven by *EML4-ALK* or *TPM3-ROS1* fusion independently. Further analysis of arm-level copy number variations (CNVs) showed consistent result with no shared CNV events between the two lesions (Table 1).

**Table 1:** Genetic alterations identified in the two primary malignancies.

**Genes**	**Alternations**	**Nucleotide change**	**Mutant allele frequency**	
			**Right ADC**	**Left ADC**
*ALK*	*EML4-ALK* fusion	–	7.5%	–
*DLL3*	p.G280R	c.838G>A	1.1%	–
*TP53*	p.R342 truncation	c.1024C>T	1.4%	–
Chr 15q		–	Amplification	–
*ROS1*	*TPM3-ROS1* fusion	–	–	10.8%
*DOT1L*	Intron 19 splice site mutation	c.1924-2A>C	–	2.5%
*SPRED1*	p.M417V	c.1249A>G	–	1.4%
Chr 4q	–	–	–	Amplification

–, *not applicable; ADC, adenocarcinoma*.

**Figure 2:**
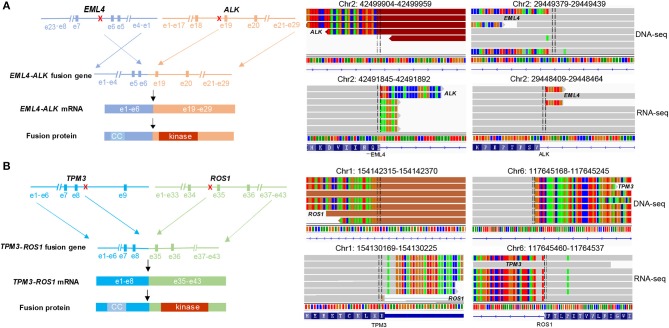
Concomitant *EML4-ALK* fusion or *TPM3-ROS1* fusion were found in each ADC. **(A,B)** Showed the fusion breakpoints detected by targeted NGS and alterations in gene, mRNA and protein level caused by two fusions, respectively. NGS sequencing reads indicating fusion regions were demonstrated by Integrative Genomics Viewer (IGV) software. CC, coiled-coil domain.

To further study the specific fusion site of *EML4-ALK* and *TPM3-ROS1* at DNA level, sequencing reads were examined on Integrative Genomic Viewer (IGV) software. The results showed that intron 5 of *EML4* fused to intron18 of *ALK* (Figure 2A), and intron 7 of *TPM3* was ligated to intron 34 of *ROS1* (Figure 2B). RNA-seq was conducted to further confirm the expression of *EML4-ALK* and *TPM3-ROS1* fusions at mRNA level (Figure 2). These two fusion proteins contain the N-terminal coiled-coil domain of the 5′ partner gene and the C-terminal tyrosine kinase domain of ALK or ROS1, which could mediate the full kinase function and result in constitutive kinase activity and oncogenic transformation (Figure 2).

During the 12 months of post-surgery follow-up (until August 2019), no recurrence or metastasis was observed in the patient.

## Disscussion

Gene fusions can be detected using a variety of techniques, including IHC (immunohistochemistry) (7), FISH (fluorescence *in situ* hybridization) (8), RT-PCT (reverse transcriptase polymerase chain reaction) (9), and NGS (10). Although *ROS1* break-apart FISH and ALK IHC are currently considered the “gold standards” in many pathology labs, NGS testing has exhibited great utility due to its comprehensiveness, high throughput and sensitivity. It also allows the identification of actual fusion partners as well as novel fusions. In a comparison study of *ALK* fusion detection by three different methods, NGS showed a comparable positive rate as IHC (92.7 and 94.5%, respectively) with the highest concordance rate. Additionally, NGS positive may indicate clinical benefit of crizotinib more accurately and provided information for concurrent genomic alterations, thereby facilitated the treatment decision making for cancer patients (11). In this case study, 425 cancer-relevant genes and potential fusions were tested simultaneously for each sample. Well-documented fusion variant of *EML4-ALK* and *TPM3-ROS1* were identified in separate tumors of the same patient, which were further confirmed negative for other known driver mutations in lung cancer.

When multiple synchronous tumors are identified in the same patient, it is always challenging to distinguish whether they are independent primary tumors or some of them are metastases from the primary site, which may have different clinical outcomes. Inferred by both gene mutation and CNV analysis, we were able to confirm that the two bilateral lung lesions are independent primary tumors in this patient. Our case serves as an example that comprehensive genomic profiling illustrated unique genomic alternations of the bilateral primary tumors, which further suggests that genetic testing for each of the multiple primary tumors may be necessary for fully molecular diagnosis and treatment decision-making.

Tyrosine kinase inhibitors (TKIs) have significantly improved the clinical outcomes of lung cancer patients over the past decade. Both *ALK* and *ROS1* fusions are targetable oncogenic fusion kinase in lung cancer (12). Due to the structural similarity of their kinase domains, crizotinib was approved worldwide for treating either *ALK* fusion-positive or *ROS1*-rearranged lung cancer. They normally appear in a mutually exclusive pattern as a driver, and so far, only three cases have been reported with *ALK*/*ROS1* double rearrangements within the same tumor with limited clinical information for their treatment strategy and patient outcome (4–6). One 77-year-old female never-smoker patient harboring concurrent *ALK/ROS1* fusions was treated with crizotinib for 3 months and responded well (5). No further follow-up information was available. Our case is the first report of a lung cancer patient with synchronous bilateral primary ADCs driven by *EML4-ALK* or *TPM3-ROS1* fusion independently. The patient was diagnosed at early stage, and the tumors were able to be surgically resected. Although no recurrence at the primary sites or metastasis has been observed during the 12-month post-surgery follow-up, it is possible that both of the fusions or only one of the fusions will contribute for the relapse later on. In either case, it is possible that the patient could be benefit from crizotinib as a second-line treatment. Additionally, since early stage resectable *EGFR* positive patients have shown benefit from neoadjuvant/adjuvant TKI treatment (13), it is possible that ALK/ROS1 positive patients could also benefit from neoadjuvant/adjuvant therapy. Our findings serve as critical information for patient's future treatment decision-making.

## Data Availability Statement

All datasets generated for this study are included in the manuscript.

## Ethics Statement

This study was approved by Ethics Committee of The Second Affiliated Hospital of Chongqing Medical University and the patient provided the written informed consent for the publication of the case report.

## Author Contributions

BT, XJ, and ZY conceptualized and designed the entire study. CT, LX, and XY carried out patient clinical management and sample collection. RW analyzed the data. BT, XJ, and RW wrote the manuscript. SL and XW revised the manuscript. All authors read and approved the final version of manuscript for submission.

### Conflict of Interest

RW and SL are the employees of Nanjing Geneseeq Technology Inc., China; XW is the employee of Geneseeq Technology Inc., Canada. The remaining authors declare that the research was conducted in the absence of any commercial or financial relationships that could be construed as a potential conflict of interest.
